# An In Vitro Comparison of Epoxy Resin Sealer Removal During Endodontic Retreatment

**DOI:** 10.7759/cureus.91536

**Published:** 2025-09-03

**Authors:** Prashant A Bondarde, Aditi S Patkar, Aishwarya R Pawar, Rukmini Pande, Akshata Deshpande, Rachana S Agrawal, Seema Gupta

**Affiliations:** 1 Department of Pedodontics and Preventive Dentistry, Jawahar Medical Foundation’s Annasaheb Chudaman Patil Memorial Dental College, Dhule, IND; 2 Department of Orthodontics, Kothiwal Dental College and Research Centre, Moradabad, IND

**Keywords:** cone-beam computed tomography, endodontic, epoxy resin-based root canal sealer, remnant, retreatment

## Abstract

Background

Endodontic retreatment involves the removal of root canal filling materials to address treatment failure or persistent infections. The success of retreatment depends on effective clearing of sealers and gutta-percha, which varies based on sealer properties and canal anatomy, especially in the apical region. This in vitro study evaluated the retreatment efficacy of three epoxy resin-based sealers in single-canal premolars, focusing on the residual sealer on dentinal walls and the time required for retreatment.

Methodology

A total of 33 extracted single-canal premolars were collected, decoronated to a standardized 17 mm length using a diamond disc, and prepared using a high-speed airotor with an endodontic access bur. Canal patency was established with a size 10 K-file, and cleaning and shaping were performed with the rotary file system up to #25/04, with irrigation using 5.25% sodium hypochlorite and 17% ethylenediamine tetraacetate gel. The teeth were divided into three groups, each having 11 teeth and obturated with gutta-percha (NeoEndo, Haryana, India), AH Plus (Dentsply, Konstanz, Germany), Sealmax-R (MAARC, India), or Epoxidin (TehnoDent, Belgrade, Serbia) using a thermoplasticizing technique. Retreatment was performed using a single retreatment file, and the times were recorded using a digital chronometer. Cone-beam computed tomography was used to assess the sealer remnants.

Results

All sealers showed comparable remnant scores in the coronal (p = 0.553) and middle (p = 0.093) thirds, but AH Plus exhibited greater apical retention (p = 0.153), leading to a higher overall score (1.48 ± 0.38) compared to Sealmax-R (1.27 ± 0.25) and Epoxidin (1.12 ± 0.17; p = 0.038). Post-hoc analysis confirmed significant differences between AH Plus and Epoxidin (mean difference = 0.36, p = 0.031). Retreatment times were the longest for AH Plus (mean = 5.97 ± 1.06 minutes), followed by Sealmax-R (mean = 5.30 ± 0.66 minutes), with Epoxidin requiring the least time (mean = 4.34 ± 0.61 minutes; p = 0.001). Apical retention was consistent across groups, suggesting anatomical challenges.

Conclusions

Epoxidin offered superior retreatability compared to AH Plus and Sealmax-R, likely due to reduced adhesive tenacity, making it preferable for anticipation of retreatment. The strong adhesion of AH Plus complicated its removal, while Sealmax-R balanced sealing and retreatability. Apical anatomy universally affected debridement, emphasizing the need for advanced retreatment techniques.

## Introduction

Endodontics, a specialized branch of dentistry, focuses on the anatomy, physiology, and pathology of dental pulp and periradicular tissues [1]. Root canal treatment is a cornerstone of endodontic practice that aims to eliminate bacteria and their byproducts through meticulous instrumentation, chemical disinfection, and obturation with biocompatible materials to restore or maintain periradicular health [2]. Effective obturation is critical for creating a fluid-tight barrier to shield periradicular tissues from oral microbes [3]. While a perfect hermetic seal is unattainable, sealers used alongside core materials such as gutta-percha play a vital role in filling irregularities, sealing lateral canals, and providing antibacterial properties, thus enhancing treatment outcomes [3].

Resin-based sealers, such as epoxy and methacrylate systems, have gained prominence because of their superior adhesion to root canal dentin compared to traditional zinc oxide eugenol or glass ionomer sealers [4]. However, endodontic failure, often marked by persistent pain or periapical lesions, can occur because of missed canals, complex root anatomy, or coronal leakage [5]. Oval-shaped canals, prevalent in approximately 25% of teeth and over 50% of incisors and premolars, pose challenges for complete disinfection, often leaving residual debris or microbial biofilms in inaccessible areas [6].

Non-surgical endodontic retreatment addresses these failures by removing obturating materials, followed by thorough cleaning, shaping, and re-obturation to eliminate necrotic tissue and persistent pathogens [2]. The effective removal of gutta-percha and sealers is crucial for successful disinfection. Various techniques, including chemical (such as chloroform and orange oil), mechanical (such as rotary nickel-titanium as Ni-Ti files), physical (such as ultrasonic and laser), and combined methods, have been employed for this purpose [7,8]. Ni-Ti rotary files are preferred because of their efficiency, safety in curved canals, and faster material removal than hand instruments [9]. Recent advancements have introduced single-file Ni-Ti systems that offer improved mechanical properties and the ability to access more canal walls, making them promising for retreatment [1,10].

Cone-beam computed tomography (CBCT) has emerged as a valuable tool in endodontics, offering three-dimensional imaging to assess treatment outcomes and guide retreatment strategies. CBCT’s superior accuracy over conventional radiographs makes it ideal for evaluating residual obturating materials after retreatment [11]. This study aimed to evaluate and compare the remnants of three resin-based sealers (AH Plus, Sealmax-R, and Epoxidin) on dentinal walls after removal using a single retreatment file, assessed via CBCT. The objectives of this study were to assess the remnants of each sealer individually using a single retreatment file and CBCT, compare the remnants of the three sealers, and measure the time required for sealer removal using a digital chronometer.

## Materials and methods

This study was designed as an in vitro experimental investigation conducted at the Department of Pedodontics and Preventive Dentistry, Jawahar Medical Foundation’s Annasaheb Chudaman Patil Memorial Dental College, Dhule, India. The study duration spanned from January 2023 to June 2023. Ethical approval was obtained from the Institutional Ethical Committee before the initiation of the study, ensuring compliance with the ethical standards for research involving human-derived samples (approval number: EC/INST/2022/2959/2022/PD12). Informed consent was obtained from the parents of the children whose extracted teeth were used, adhering to the ethical guidelines for the use of biological materials in research.

The sample size was determined using the G*Power software (version 3.6.9.2, Heinrich Heine University, Düsseldorf, Germany). Based on an effect size of 0.58 (derived from prior research measuring the remaining AH Plus sealer in retreatment cases), a minimum of 33 total samples was required to achieve 80% statistical power with a 5% alpha error level [12]. As the study comprised three experimental groups, 11 samples were allocated to each group to maintain balanced group sizes.

This study included 33 single-canal premolar teeth that were selected based on specific inclusion and exclusion criteria to ensure sample homogeneity. The inclusion criteria were teeth with sufficient root length, single-canal premolar teeth, and roots with a closed apex. The exclusion criteria were teeth with morphological variations, fractured roots, cervical abrasion, root surface caries, grossly carious teeth, or calcified canals.

Each tooth was thoroughly washed under running tap water for two minutes to remove debris and then immersed in a 10% formalin solution for seven days to ensure disinfection. Any calculi on the root surfaces were removed using a hand-scaling instrument (GDC; Hoshiarpur, Punjab, India). The prepared teeth were stored in a normal saline solution (Aculife, India) at 37°C and 95% humidity to mimic physiological conditions until further processing.

**Figure 1:**
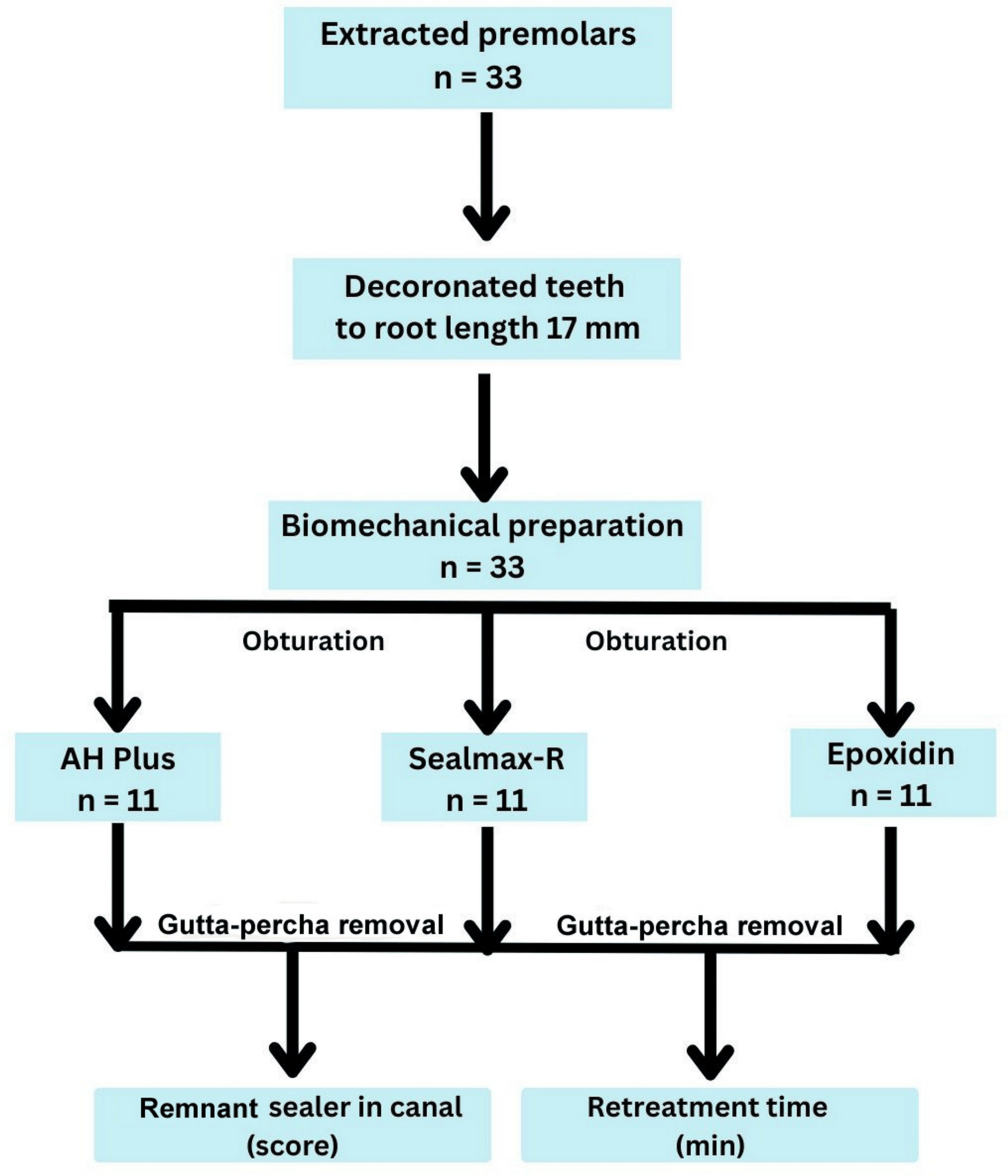
Study flowchart.

For root canal preparation, the teeth were removed from saline solution and air-dried on absorbent paper. Each tooth was decoronated at the cementoenamel junction using a diamond disc (Mani Inc., Tochigi, Japan) to achieve a standardized root length of 17 mm across all samples. Conventional root canal access was performed using a high-speed airotor handpiece (NSK, Japan) with a round endodontic access burr (BR41, Mani Inc., Tochigi, Japan). Canal patency was confirmed by inserting a size 10 K-file (Mani Inc., Tochigi, Japan) until it was visible at the apical foramen, and the working length was established by subtracting 0.5 mm from this measurement. To prevent irrigant extrusion, the apices of all the teeth were sealed with nail varnish. Root canal cleaning and shaping were performed using a rotary file system (NeoEndo Gold, Orikam Healthcare India Pvt. Ltd., Haryana, India) up to size 25/04, following the manufacturer’s instructions. During this process, constant irrigation was maintained with 5.25% sodium hypochlorite (Cerkamed Chloraxid Extra, Poland) using an irrigation needle (Irriflex, Produits Dentaires, Switzerland) and 17% ethylene diamine tetraacetate gel (Prime Dental RC Help, India) as a lubricant. After cleaning and shaping the canals, the canals were rinsed with normal saline (Aculife, India). The teeth were then randomly divided into three groups of 11 teeth each for obturation with different sealers (Figure 1).

The three groups were assigned as follows: Group 1 (n = 11) used AH Plus resin sealer (Dentsply, Konstanz, Germany), Group 2 (n = 11) used Sealmax-R resin sealer (MAARC, India), and Group 3 (n = 11) used Epoxidin resin sealer (TehnoDent, Belgrade, Serbia). For obturation, the root canals were dried using #25/04 absorbent paper points (NeoEndo, Haryana, India). In Group 1, the AH Plus sealer was prepared by mixing equal volumes of Paste A and Paste B on a glass slab with a metal spatula (Mani Inc., Tochigi, Japan) until homogeneous consistency was achieved. A #25/04 gutta-percha point (NeoEndo, Haryana, India) was coated with a sealer and applied to the canal walls using a pumping and rotating motion, followed by obturation with a thermoplasticized technique using an obturation device (Fast Fill 3D, Eighteeth, Orikam, India).

**Figure 2:**
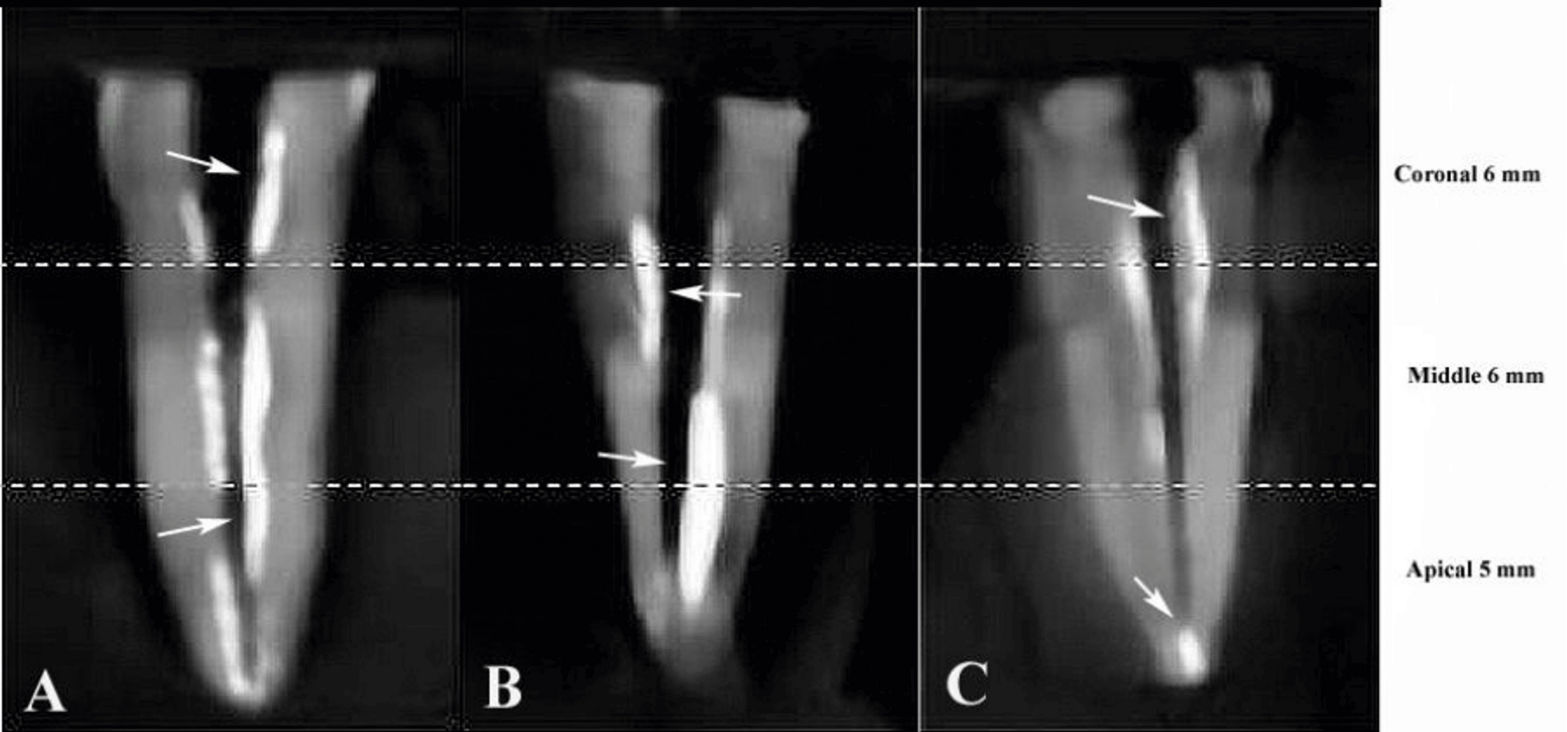
Remnants of sealer on dentinal wall shown by cone-beam computed tomography (CBCT) images of root canal at various levels. (A) AH Plus. (B) Sealmax-R. (C) Epoxidin. Original CBCT images of the teeth in the study.

In Group 2, the Sealmax-R sealer was prepared by mixing one scoop of powder with one drop of liquid on a mixing pad using a plastic spatula until a homogeneous consistency was achieved. The sealer was applied to the canal walls with a #25/04 gutta-percha point, avoiding air bubbles, and was obturated using the thermoplasticized technique. In Group 3, the Epoxidin sealer was prepared by mixing white and yellow pastes in a 1:1 ratio on a glass slab with a metal spatula until uniform color and consistency were obtained. The sealer was applied to the canal walls with a #25/04 gutta-percha point, followed by thermoplasticized obturation. All samples were embedded in putty rubber base material (Zhermack, Badia Polesine, Italy) and subjected to CBCT scanning using a CBCT machine (Planmeca PROMAX 3D. Planmeca, Helsinki, Finland) with Romexis software, operating at 90 kV, 6.3 mA, 12.064, and 0.200 mm axial slice thickness to evaluate voids in the obturation. Subsequently, all samples underwent composite restoration (Composite Z350, 3M, St. Paul, MN, United States) and were stored at 37°C for three weeks (Figure 2).

For the retreatment phase, the composite restorations were removed, and retreatment was performed using a single retreatment file system (#25/06, RETREAT, Micromega, Besançon, France). The time required for retreatment was recorded using a digital chronometer (VWR Int., Pennsylvania, US). A second CBCT scan was performed to assess residual sealer on the dentinal walls. Sealer remnants were evaluated by a single pre-calibrated, blinded operator using direct visual scoring of CBCT images, based on a grading system adapted from Uzunoglu et al. [13]. The scoring criteria were as follows: 1 (0-25% debris on dentin surfaces), 2 (25-50% debris), 3 (50-75% debris), and 4 (>75% debris). The root canals were divided into coronal (6 mm), middle (6 mm), and apical (5 mm) thirds, and the sealer residues in each portion were scored and compared according to established criteria.

All procedures were standardized to ensure reliability, and the operator performing the CBCT evaluation was pre-calibrated to minimize bias. The single-blinded approach further enhanced the objectivity of the sealer remnant assessment. The use of a standardized grading system and precise division of the canal thirds ensured a consistent evaluation across samples. The study’s methodology was designed to maintain reproducibility, with all instruments and materials sourced from reputable manufacturers, and their specifications, including company headquarters, clearly documented to ensure the traceability and reliability of the results.

Data were analyzed using SPSS software version 2020 (IBM Corp., Armonk, NY, USA). The data were checked for normality using the Kolmogorov-Smirnov test and found to be normally distributed. Sealer scores (ordinal data) were presented as the median, mean, and standard deviation. Comparison of the mean sealer remnant scores between study groups was performed using the Kruskal-Wallis test for ordinal data (score) and one-way analysis of variance (ANOVA) for continuous data (such as retreatment time). Pairwise comparisons between groups were performed using the Bonferroni test and the Tukey HSD post-hoc test. Sealer scores within the group at multiple locations (coronal, middle, and apical) were compared using the Friedman test. The level of significance was kept at 5% (p-value).

## Results

Descriptive analysis revealed comparable sealer remnant scores across the groups in the coronal and middle thirds. AH Plus showed slightly higher apical retention, yielding a higher overall score (1.33) and mean rank (20.73). Sealmax-R and Epoxidin performed similarly. Although all sealers demonstrated effective removal, the marginally inferior apical clearance of AH Plus may reflect its higher adhesion in complex anatomies (Table 1).

**Table 1:** Descriptive analysis of distribution of sealer remnant scores in groups. Scores represent the median amount of sealer remnant in each root canal section. The overall score is the mean of the median scores across sections. Mean rank is derived from the Kruskal-Wallis test for intergroup comparison. Distribution of teeth in each group is represented as frequency (n) and percentage (%), where n denotes the number of teeth.

Groups	n (%)	Coronal (6 mm) (median score)	Middle (6 mm) (median score)	Apical (5 mm) (median score)	Overall score	Mean rank
AH Plus	11 (33.33)	1	1	2	1.33	20.73
Sealmax-R	11 (33.33)	1	1	1	1.00	16.55
Epoxidin	11 (33.33)	1	1	1	1.00	13.73

Kruskal-Wallis analysis revealed no significant differences in the coronal (p = 0.553), middle (p = 0.093), or apical (p = 0.153) sealer remnant scores between the groups. However, AH Plus showed significantly higher overall scores (1.48 ± 0.38) than Sealmax-R (1.27 ± 0.25) and Epoxidin (1.12 ± 0.17; p = 0.038). While all groups performed comparably at the individual root levels, the cumulative retention of AH Plus suggested material-specific differences in complete canal debridement (Table 2).

**Table 2:** Intergroup comparison of sealer remnant scores across different regions of root levels. *: P < 0.05 denotes statistical significance using the Kruskal-Wallis test. The sealer remnant scores are presented as mean ± standard deviation (SD).

Area of assessment	AH Plus	Sealmax-R	Epoxidin	Test statistics	P-value
	Mean ± SD	Mean ± SD	Mean ± SD		
Coronal (6 mm)	1.27 ± 0.47	1.18 ± 0.41	1.09 ± 0.30	1.19	0.553
Middle (6 mm)	1.36 ± 0.51	1.18 ± 0.41	1.00 ± 0.01	4.74	0.093
Apical (5 mm)	1.82 ± 0.75	1.45 ± 0.52	1.27 ± 0.47	3.76	0.153
Overall	1.48 ± 0.38	1.27 ± 0.25	1.12 ± 0.17	6.55	0.038*

Post-hoc analysis revealed significant differences only between AH Plus and Epoxidin (mean difference = 0.36, adjusted p = 0.031). This indicates that AH plus sealer removal efficacy was superior to that of the other groups (Table 3).

**Table 3:** Pairwise comparison of groups for overall sealer remnant scores using post-hoc analysis. *: Adjusted p < 0.05 denotes statistical significance using the Bonferroni post-hoc test. Mean difference: Difference of overall scores between paired groups, where scores of the second group are subtracted from the first group.

Pairwise	Mean difference	Test statistic	Standard test statistic	P-value	Adjusted p-value
AH Plus vs. Sealmax-R	0.21	4.95	1.28	0.201	0.602
AH Plus vs. Epoxidin	0.36	9.91	2.56	0.01	0.031*
Sealmax-R vs. Epoxidin	0.15	4.95	1.28	0.201	0.602

Friedman test analysis revealed no significant differences in the sealer remnant scores across the coronal, middle, and apical regions within any group. Although all the groups showed numerically higher scores apically, these trends were not statistically significant. The consistent apical accumulation pattern across the materials suggests potential anatomical challenges during debridement (Table 4).

**Table 4:** Within group comparison of sealer remnant scores obtained from different locations of root. P > 0.05 denotes no statistical significance using the Friedman test. The sealer remnant scores are presented as mean ± standard deviation (SD).

Groups	Coronal (6 mm)	Middle (6 mm)	Apical (5 mm)	Test statistics	P-value
	Mean ± SD	Mean ± SD	Mean ± SD		
AH Plus	1.27 ± 0.47	1.36 ± 0.51	1.82 ± 0.75	2.36	0.307
Sealmax-R	1.18 ± 0.41	1.18 ± 0.41	1.45 ± 0.52	1.23	0.541
Epoxidin	1.09 ± 0.30	1.00 ± 0.001	1.27 ± 0.47	0.95	0.621

One-way ANOVA revealed significant differences in retreatment time between the groups (p = 0.001). Post-hoc analysis showed that AH Plus required a significantly longer time than Epoxidin (mean difference = 1.63 minutes, p = 0.001), while Sealmax-R was intermediate, but still significantly required a longer time than Epoxidin (mean difference = 0.96 minutes, p = 0.023) (Table 5).

**Table 5:** Comparison of retreatment time (minutes) required in study groups with one-way analysis of variance (ANOVA) followed by Tukey post-hoc analysis. *: P < 0.05 denotes statistical significance using one-way ANOVA, with pairwise comparisons by the Tukey post-hoc test. Data are presented as mean ± standard deviation (SD). Mean difference: difference of retreatment time (minutes) between paired groups, where retreatment time of the second group was subtracted from the first group.

Groups	Retreatment time (minutes)	F value	P-value	Pairwise comparison	Mean difference (minutes)	P-value
AH Plus	5.97 ± 1.06	11.46	0.001*	AH Plus vs. Sealmax-R	0.67	0.140
Sealmax-R	5.30 ± 0.66			AH Plus vs. Epoxidin	1.63	0.001*
Epoxidin	4.34 ± 0.61			Sealmax-R vs. Epoxidin	0.96	0.023*

## Discussion

The results of the present study indicated that sealer remnant scores were comparable in the coronal and middle thirds across all groups, suggesting that these regions pose fewer challenges for debridement. Wider canal diameters and better instrument accessibility in these areas are likely to facilitate effective mechanical instrumentation and irrigation, enabling consistent sealer removal. This observation aligns with previous studies, which noted that sealer removal is generally more straightforward in the coronal and middle canal portions owing to anatomical advantages and enhanced instrument contact [13,14]. The lack of significant differences in these regions across the tested sealers indicated that their chemical composition and physical properties had a limited impact on retreatability in more accessible canal segments. This is likely applicable to Adseal, Proseal, and AH26, as their epoxy resin bases provide similar flow and adaptability to canal walls, as noted in a study by Saeidi et al. [15], who found comparable apical sealing between Adseal, Proseal, and AH26.

However, AH Plus exhibited greater retention in the apical third than did Sealmax-R and Epoxidin, leading to a higher overall sealer remnant score. This suggests that AH Plus’s epoxy resin-based composition may result in stronger adhesion to dentin or deeper penetration into dentinal tubules, particularly in the apical region, where canal anatomy is often complex, with narrower diameters and potential irregularities, such as accessory canals or isthmuses. The adhesive strength of AH Plus has been well-documented in the literature. For example, Almeida et al. [16] highlighted its excellent bonding to dentin, which is attributed to the ability of epoxy resin to form a robust chemical bond with the dentinal surfaces. Pawar et al. [17] noted the lowest strength was noted in oval canals instrumented with WaveOne and filled with gutta-percha and AH Plus sealer. While this property of AH Plus adhesion enhances sealing during initial obturation, it appears to complicate retreatment because the tenacity of the sealer in the apical region hinders complete removal [18]. The anatomical constraints of the apical third, such as reduced irrigant penetration and limited instrument contact, likely exacerbate this challenge.

In contrast, Epoxidin demonstrated the lowest overall sealer remnant score, indicating superior retreatability compared to AH Plus, with post-hoc analysis confirming a significant difference. This suggests that Epoxidin formulation, despite being epoxy resin-based, may include additives or curing properties that reduce its adhesive tenacity, allowing easier mechanical disruption during retreatment. Although specific studies on Epoxidin are scarce, research on other modified resin-based sealers, such as Song et al. [19], suggests that variations in resin composition can influence the ease of removal. Sealmax-R performed intermediately, showing no significant difference from Epoxidin but better clearance than AH Plus in the apical region. Its powder-liquid formulation may result in a less cohesive bond compared to AH Plus’s paste-based system, facilitating moderately easier removal while maintaining adequate sealing properties. Hwang et al. [20] observed lower leakage scores with the AH26 sealer than with the Sealmax-R sealer. The disparity in the results could be due to differences in the methodology, as the authors used the dye penetration method.

The significant differences in the retreatment times further highlight material-specific variations. AH Plus required the longest retreatment time, followed by Sealmax-R and Epoxidin. This aligns with AH Plus’s higher apical retention, as its stronger adhesion likely necessitates more extensive mechanical effort to dislodge [12]. Similarly, Hess et al. [21] noted that epoxy resin-based sealers often require additional instrumentation time, owing to their robust dentin adhesion. The shorter retreatment time of Epoxidin suggests a less resistant material structure, enabling a single retreatment file to remove it more efficiently. The intermediate performance of Sealmax-R may reflect its unique preparation method, where powder-liquid mixing could produce a less cohesive sealer mass than AH Plus, allowing moderately faster removal.

The absence of significant differences in sealer remnant scores across the coronal, middle, and apical thirds within each group, as determined by the Friedman test, indicated that the trend toward higher apical accumulation was consistent across all sealers. This pattern likely reflects anatomical challenges, rather than solely the material properties. The complex morphology of the apical third, including narrower canals and potential ramifications, limits the efficacy of rotary instruments and irrigants [12]. This consistent apical accumulation suggests that the choice of retreatment instruments and irrigation protocols is critical for overcoming the anatomical barriers. The significant overall difference in sealer remnant scores, particularly between AH Plus and Epoxidin, underscores the importance of sealer selection in cases where retreatment is anticipated. The ANOVA results for retreatment time further highlight that material properties affect clinical efficiency, with faster retreatment times potentially reducing chair time and improving the patient experience.

The retreatment procedure in this study utilized a single retreatment file system to remove root canal filling materials, followed by a thermoplasticized condensation technique for obturation. This file, designed specifically for retreatment, features unique cutting efficiency and flexibility, allowing the effective removal of gutta-percha and sealer from the canal walls, particularly in the complex apical region. Its single-file approach simplifies the procedure and reduces instrumentation time while maintaining efficacy. Owing to the lack of literature on this file system, we supported our results with a file system similar to that documented by Singh et al. [22]. For condensation, the thermoplasticized technique enhances sealer flow into canal irregularities, improving adaptation and sealing [23], although it may contribute to increased apical retention, particularly for adhesive sealers, such as AH Plus.

Clinical implications

These findings have significant implications in clinical endodontics, particularly in retreatment scenarios. The superior retreatability of Epoxidin makes it a preferable choice for cases in which future retreatment is likely, such as teeth with a higher risk of persistent infection or complex canal anatomy. A shorter removal time could enhance clinical efficiency, reduce procedural duration, and cause patient discomfort. Conversely, the strong adhesion of AH Plus, which is ideal for long-term sealing in primary root canal treatments, may complicate retreatment, requiring more time and potentially requiring additional instrumentation or solvents. Clinicians should consider these factors when selecting a sealer to balance the need for effective sealing with the potential for future intervention. The intermediate performance of Sealmax-R offers a balanced option suitable for cases requiring both reliable sealing and reasonable retreatability. The consistent challenge of apical sealer retention across all groups highlights the need for advanced irrigation techniques, such as ultrasonic activation or laser-assisted irrigation to enhance debridement in the apical third, where anatomical complexities pose significant barriers.

Limitations

This study has several limitations. First, its in vitro design may not fully replicate the clinical environment, where factors such as periapical tissue, saliva, and patient-specific anatomical variations may influence the retreatment outcomes. Second, the use of single-canal premolars limits generalizability to multirooted teeth or those with more complex canal systems. Third, the evaluation relied on a single blinded operator, which, while reducing bias, may have introduced subjectivity in the visual scoring of the CBCT images. Additionally, this study did not explore the impact of different retreatment instruments or irrigation protocols that could modulate the efficacy of sealer removal. Finally, the lack of specific data on Epoxidin composition limits its ability to fully explain its superior retreatability, warranting further research into its material properties.

## Conclusions

This study demonstrated that the retreatment efficacy of epoxy resin-based sealers varies, with Epoxidin exhibiting superior ease of removal compared to AH Plus and Sealmax-R, particularly in the apical region, likely owing to differences in adhesive properties and material composition. AH Plus showed greater retention, requiring longer retreatment times, while Sealmax-R performed intermediately. The consistent challenge of apical sealer accumulation across all groups highlights the influence of the canal anatomy on debridement outcomes. These findings suggest that sealer selection should consider the potential need for retreatment, with Epoxidin offering advantages when efficient removal is prioritized.
